# Radiationless anapole states in on-chip photonics

**DOI:** 10.1038/s41377-021-00647-x

**Published:** 2021-10-04

**Authors:** Evelyn Díaz-Escobar, Thomas Bauer, Elena Pinilla-Cienfuegos, Ángela I. Barreda, Amadeu Griol, L. Kuipers, Alejandro Martínez

**Affiliations:** 1 Nanophotonics Technology Center, Universitat Politècnica de València, Camino de Vera s/n, 46022 Valencia, Spain; 2 Department of Quantum Nanoscience, Kavli Institute of Nanoscience, Delft University of Technology, 2600 GA Delft, The Netherlands; 3 Institute of Applied Physics, Abbe Center of Photonics, Friedrich Schiller University Jena, Albert-Einstein-Straße 15, 07745 Jena, Germany

**Keywords:** Integrated optics, Microresonators, Silicon photonics, Nanophotonics and plasmonics

## Abstract

High-index nanoparticles are known to support radiationless states called anapoles, where dipolar and toroidal moments interfere to inhibit scattering to the far field. In order to exploit the striking properties arising from these interference conditions in photonic integrated circuits, the particles must be driven in-plane via integrated waveguides. Here, we address the excitation of electric anapole states in silicon disks when excited on-chip at telecom wavelengths. In contrast to normal illumination, we find that the anapole condition—identified by a strong reduction of the scattering—does not overlap with the near-field energy maximum, an observation attributed to retardation effects. We experimentally verify the two distinct spectral regions in individual disks illuminated in-plane from closely placed waveguide terminations via far-field and near-field measurements. Our finding has important consequences concerning the use of anapole states and interference effects of other Mie-type resonances in high-index nanoparticles for building complex photonic integrated circuitry.

## Introduction

Light scattering by high-refractive-index dielectric nanoparticles can be explained by the formation of a set of radiative displacement currents upon illumination with an external source^1,2^. Under certain conditions, a given combination of displacement currents may not scatter light due to destructive interference in the far field, resulting in the so-called anapole state^3–10^. Describing the electromagnetic response of the nanoparticle by a set of multipole eigenstates (derived from the polarization currents), such an anapole condition results from the antiphase oscillation of the Cartesian electric and toroidal dipole or corresponding higher-order multipoles^3–10^. Notably, an anapole is not a natural eigenmode of a certain electromagnetic structure so that, for example, it cannot lead to lasing^9^. In contrast, an anapole is a distinct state that results from the interference between two eigenmodes of the nanoparticle, thus being reminiscent of the first and second Kerker conditions^11^ arising from the interference between electric and magnetic dipolar Mie-type resonances, as well as the formation of accidental quasi-bound states in the continuum via the strong coupling of a Mie-type and a Fabry-Pérot-type resonance^12^.

Unlike metamaterial-based cloaking structures^13^, which are characterized by the complete absence of local internal fields^9,14^, anapole states are usually accompanied by a strong concentration of electromagnetic energy inside the nanoparticle^10,15^. This remarkable feature has been used to boost light-matter interaction and enhance nonlinear effects such as harmonic generation^16–18^ or Raman scattering^19^ in different experiments.

A prototypical structure to achieve these radiationless anapole states is a high-index nanodisk^3,4,15^, which can be easily fabricated on low-index substrates using standard lithography techniques. The two main signatures of the electric anapole condition in high-index disks are: (i) in the far field, a strong suppression of scattering^3^ and (ii) in the near field, the formation of poloidal displacement currents with the electric field forming local vortices, with opposite rotation sense in each half of the disk^15^. To the best of our knowledge, all previous experiments reporting on the observation of anapole states have made use of free-space light impinging on the disks in a direction perpendicular to the substrate (out-of-plane incidence), with the disk either flat on the substrate^3^ or vertically standing^18^. However, it would be highly interesting to look at the properties of such states when excited by light propagating in-plane, as is the case in photonic integrated circuits (PICs), which are extensively used in multiple applications, including optical processing and communications, sensing or spectroscopy^20^. In this sense, the realization of anapole states may add novel functionalities to the existing utilization scenarios of high-index disks in PICs^21,22^. In particular, anapole states could play a key role in nonlinear light processing in PICs, for instance, by reducing the footprint and power consumption of all-optical switching^23^ and other nonlinear processing elements^24^ while the undesired out-of-plane scattering could be completely suppressed. In comparison with other subwavelength-sized resonators, such as photonic crystal cavities, anapoles display a much broader bandwidth, which would enable a faster response as well as supporting processes such as four-wave mixing^25^, which could be employed in nonlinear signal processing.

In this work, we analyze both numerically and experimentally the in-plane realization of an anapole state in individual silicon disks driven by the fundamental TE-like mode of a silicon waveguide, so that the incident light travels normal to the disk axis. We use numerical simulations to study the near-field patterns, as well as the far-field scattering under both out-of-plane and in-plane illumination. We calculate the multipolar moments for both cases, leading to a blue-shift of the electric anapole condition (identified as the wavelength for which the electric dipole and toroidal moments cancel each other in the far field) for in-plane incidence in comparison to the usual normal incidence case. We attribute this phenomenon to retardation effects due to the wavelength-scale size of the disk along the illumination direction. More importantly, this additionally results in a decoupling of the minimum in the far-field scattering and the maximum of energy localization in the disk, which take place at well separated wavelengths for in-plane excitation. We conduct far-field experiments recording out-of-plane scattering of different individual disks (excited by waveguides) and observe a strong reduction of the scattering in the wavelength region where the anapole condition is predicted to occur. We furthermore report on near-field scanning optical microscopy (NSOM) experiments that illustrate the typical dual-vortex near-field pattern attributed to anapole states as well as a strong energy concentration at wavelengths red-shifted to the anapole condition, in agreement with the numerical simulations. Our results highlight important differences between anapole states when the particles are illuminated from different directions and have direct implications to the use of wavelength-size disks in high-index PICs for applications ranging from biosensing to nonlinear signal processing.

## Results

### Scattering and multipolar response of silicon disks under normal and in-plane illumination

**Fig. 1:**
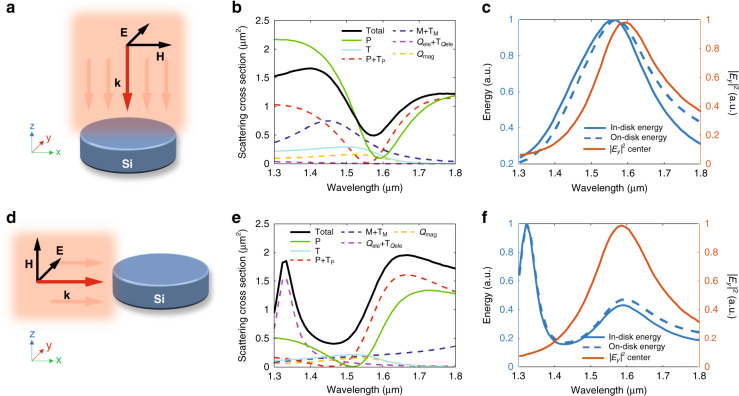
Optical response of a silicon disk in air. The direction of incidence of the incoming plane wave can be either parallel (**a**–**c**) or perpendicular (**d**–**f**) to the disk axis. The spectral contribution of the main Cartesian multipolar terms (electric dipole (**p**), magnetic dipole (**m**), electric quadrupole (*Q*_ele_), magnetic quadrupole (*Q*_mag_) and their respective toroidal moments for the electric dipole, magnetic dipole and electric quadrupole modes, represented by (T)) to the scattering cross-section is shown in **b** and **e** for each case. The total scattering cross-section is represented in black. The spectra representing the energy inside the disk and on the disk surface, as well as the electric-field intensity at the disk center for each configuration are depicted in **c** and **f**, respectively. The disk dimensions are *r* = 350 nm and *t* = 220 nm. Details about the calculations can be found in the “Methods” section

We start our study by calculating the scattering of light by a single silicon disk surrounded by air. We fix the disk thickness to *t* = 220 nm, which is the usual silicon thickness in commercial silicon-on-insulator wafers, while the radius *r* can be varied to tune the particular contribution of each multipolar moment at a certain wavelength^4^. Indeed, by properly choosing the dimensions of the disk, the destructive interference between the in-plane electric dipole and the toroidal moments can be tuned to a specific spectral region (in our case: the experimentally accessible region of *λ* = 1260 − 1630 nm for far-field measurements). We consider two directions of incidence of the incoming wave: it can be either parallel (out-of-plane excitation) or perpendicular (in-plane excitation) to the disk axis, as shown in Fig. 1a, d, respectively. For the latter, we consider that the electric (magnetic) field of the excitation wave is perpendicular (parallel) to the disk axis. The contributions to the scattering cross-section of the main multipole moments (Cartesian electric **p**, magnetic **m**, quadrupolar electric *Q*_ele_ and quadrupolar magnetic *Q*_mag_, together with their respective toroidal moments (**T**) for the case of the dipolar electric, dipolar magnetic and quadrupolar electric modes), are shown in Fig. 1b, e for out-of-plane excitation and in-plane excitation, respectively. It can be seen that the anapole condition—this is, ∣**p**∣ = ∣ − ı*k***T**∣, where *k* is the wave number—is satisfied in both configurations. However, it occurs at significantly different wavelengths (1555 nm for out-of-plane incidence, and 1444 nm for in-plane incidence), which—at first glance—is a surprising fact when we consider that the dimensions of the disk are the same and the incident electric field is normal to the disk axis in both cases. Another remarkable difference arises when looking at the energy stored in the disk, which is directly related to the intensity of the electric field. While for normal incidence, the energy and electric-field intensity maxima are observed close to the anapole condition (Fig. 1c), in agreement with previous works^3,4,16–19^, for in-plane incidence these magnitudes are red-shifted to wavelengths around 1600 nm, as depicted on Fig. 1f. This means that the absence of scattering is no longer linked to a maximization of the energy stored (or electric-field intensity) in the disk. Regarding the quadrupolar terms, although this scattering is in general smaller than the one coming from the dipolar terms, it becomes predominant around the anapole region, preventing a complete cancellation of scattering. However, in our experimental realization, we measure the scattered far field along the disk axis and in this direction the contribution from the quadrupolar terms should be negligible, thus allowing us to determine the anapole condition, as shown below.

**Fig. 2:**
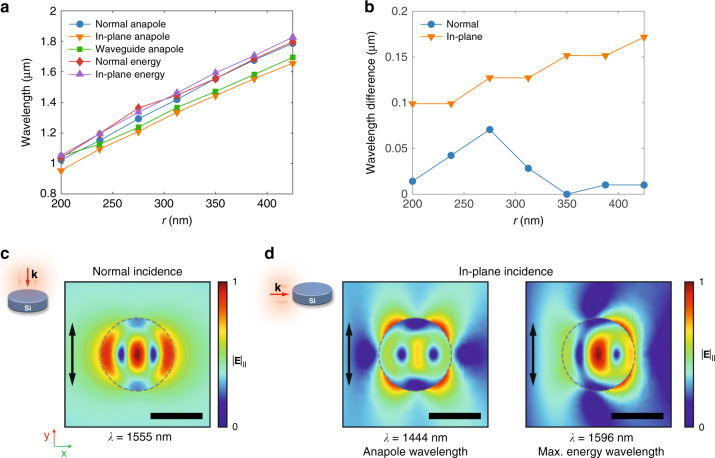
Spectral evolution of the anapole condition at normal and in-plane incidence. **a** Anapole-condition and maximum-energy wavelengths for normal and in-plane incidence as a function of the disk radius *r*. The wavelength at which a minimum of the out-of-plane scattering is observed under waveguide illumination is also shown. **b** Wavelength difference between the energy maximum and the anapole for the cases represented in **a**. Calculated electric-field amplitude patterns in a disk of *r* = 350 nm at the relevant wavelengths for both **c** normal and **d** in-plane incidence. The black arrows indicate the polarization direction of the incident electric field. Scale bar: 500 nm. The calculations have been performed using COMSOL Multiphysics (see “Methods”)

We can explain this discrepancy between energy maximum and scattering minimum by considering that the disk has a non-negligible size (the diameter is roughly half of the involved wavelength at the anapole condition) in the propagation direction for in-plane incidence. Therefore, retardation effects play a role and largely modify the response of the disk with respect to the normal incidence case^26,27^. For normal incidence (and a thin disk), we excite the in-plane eigenmodes of the disk without phase retardation between different lateral points of the disk, while for in-plane incidence the retardation of the excitation field between different lateral points in the disk means the quasi-static approximation does not hold anymore. Similar to effects shown in plasmonic particles^28,29^ the resonance frequencies will thus shift, leading to a wavelength shift of the anapole condition. We calculated the anapole and maximum-energy wavelengths for different disk radii keeping the thickness equal to 220 nm. As shown in Fig. 2a, b, the anapole and maximum-energy wavelengths almost coincide for normal excitation, except for some specific radii such as around *r* = 275 nm, probably due to the contribution of other multipolar moments to the energy storage inside the disk. However, for in-plane incidence both conditions (anapole and energy maximum) are decoupled. Moreover, the wavelength spacing between them grows proportional to the disk dimensions, which supports our argument on the retardation effects. Furthermore, we likewise observed this effect when calculating both wavelengths for disks having the same radius but different thicknesses (see Supplementary Fig. S1). Indeed, in this case the decoupling of the two conditions is also evident under normal incidence for sufficiently thick disks.

Besides these observations, some differences can additionally be seen in the near-field patterns depicted in Fig. 2c, d. For normal incidence (Fig. 2c), the typical pattern with three maxima and two nodes of the electric field (forming the expected vortices of the electric field) is observed. In the case of the anapole state under in-plane incidence (Fig. 2d), these features also appear, but the electric-field is not as well confined in the disk. Instead, we observe four external field maxima surrounding the disk at the azimuthal positions *π*/4, 3*π*/4, 5*π*/4, and 7*π*/4. When considering the field pattern at the maximum-energy wavelength, we can see that the electric field concentration inside the disk increases considerably. The two electric-field vortices are still present, but there is a strong asymmetry between them, with the first one being partly obscured by the incident field while the second one is dominating the field distribution. Still, the signature of the toroidal excitation is present in the energy maximum, as is expected due to the broadband character of this resonance.

### In-plane excitation of an on-chip silicon disk: numerical simulations

**Fig. 3:**
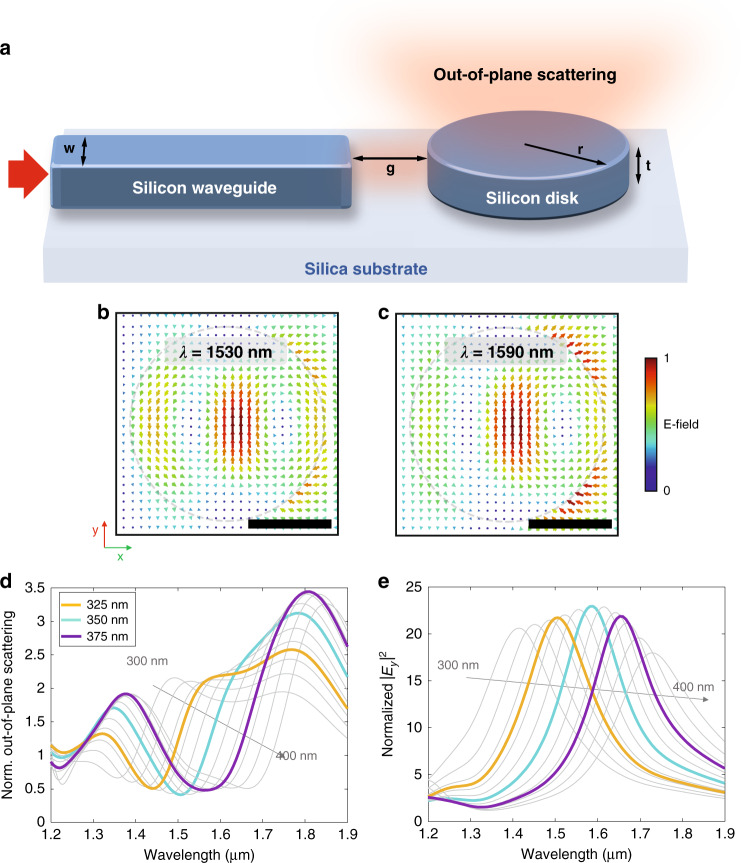
In-plane excitation of a silicon disk from a waveguide. **a** Sketch of the in-plane excitation of a silicon disk from a waveguide end. **b** Electric-field lines at the anapole and **c** maximum-energy wavelengths under waveguide illumination for a *r* = 350 nm disk with waveguide width *w* = 420 nm and gap *g* = 600 nm, with the system placed on a silica substrate. Scale bar: 300 nm. 3D-FDTD simulations of the normalized out-of-plane scattering (**d**) and the electric-field intensity ∣*E*_*y*_∣^2^ at the disk center (**e**) for disk radius *r* varying between 300 and 400 nm (325, 350, and 375 nm in color and the rest in 10 nm steps in gray as visual guide). Normalization is performed by comparing with the same case without the silicon disk

To achieve in-plane (or on-chip) excitation of the silicon disk, we consider a silicon strip waveguide of width *w* ended abruptly by a flat termination and spaced by a gap *g* from the disk boundary, both situated on a silica substrate (Fig. 3a). A similar configuration was used in previous works to excite individual plasmonic nanostructures via integrated waveguides^30,31^. Notice also that a similar waveguide-disk configuration was proposed to extract light generated internally in the disk via near-field coupling^32^, while here we choose a gap *g* = 600 nm where no significant near-field interaction between waveguide and disk remains. By coupling light to the TE-like mode of the waveguide from the left side, light exiting from the waveguide end will excite the disk mainly via an in-plane electric field *E*_*y*_ and an out-of-plane magnetic field *H*_*z*_. Note that the tight field confinement of the waveguide will also result in the existence of longitudinal components of the fields (*E*_*x*_, *H*_*x*_)^30,33,34^ that also contribute to the disk excitation because of the proximity of the waveguide termination and the disk boundary (*g* < *λ*). In principle, the contribution of such terms should be much smaller than the transversal ones, but the overall picture will be slightly different from the plane-wave case considered in the previous calculations. Still, the near-field patterns shown in the field maps in Fig. 3b, c look quite similar to the ones presented in Fig. 2d for in-plane illumination. For instance, they evidence the formation of three main lobes of the electric field, which confirms the capability of our approach to excite the anapole state. Indeed the near-field patterns look almost identical for the energy maximum wavelength while some discrepancies arise at the anapole condition wavelength. This can be explained by the presence of the substrate that breaks the system’s mirror symmetry in the vertical direction. The substrate effect is more pronounced at the anapole condition since the fields are not as strongly localized in the disk and are more perturbed due to the change of the refractive index below the disk. More details about the near-field patterns under waveguide illumination are shown in Supplementary Fig. S3 and the movies included in the Supplementary materials.

The spectral response of the disk when illuminated from a nearby waveguide termination shows similar features as in the case of free-space illumination addressed above. Figure 3d, e show the out-of-plane scattering and electric-field intensity spectra under waveguide illumination as a function of the disk radius. As expected, increasing the disk radius red shifts both wavelengths, which confirms that both the anapole condition and the energy maximum can be carefully tuned over the whole telecom wavelengths window. Additionally, these results show that the scattering minimum and the energy maximum take place at different wavelengths regardless of the radius of the disk. The calculated values have been normalized with respect to the same scenario without disk to account for the effect of the waveguide: in the case of scattering, since the field has been monitored at a distance of 2 µm from the disk top surface, we remove the effect of the evanescent field of the TE mode; in the case of the electric-field intensity in the disk center, we eliminate the effect caused by the field diverging at the waveguide output. Note that here the scattering is monitored only along the vertical axis (*θ* = 0^∘^), that is, we are calculating the out-of-plane scattering. This is a good approximation to pinpoint the anapole condition since only the electric dipole and the toroidal moment will contribute to the scattering along that direction (see Supplementary Fig. S2).

In the fabricated samples, both the waveguide and the disk need to rest on a silica substrate. We therefore considered the effect of the silica substrate on the features of the silicon disk. As shown in Supplementary Fig. S4, the presence of the substrate red shifts the anapole condition (scattering minimum in the far field), as can be expected since the refractive index of the whole system increases. However, the maximum-energy wavelength remains virtually the same, which can be understood by considering that the field is strongly confined inside the disk and does not feel the presence of the substrate. Thus, this near-field property remains unchanged when introducing a substrate with moderate refractive-index contrast to the disk. We also calculated the energy above the disk (see Fig. 1f for free-space illumination and Supplementary Fig. S4 for waveguide illumination), which shows that the energy maximum at that plane coincides with the energy maximum inside the disk. This is an important point to be considered for the application of anapole states in nanophotonic sensors, as well as for the near-field measurements detailed below, where the energy is measured on the top surface of the disk.

### Far-field scattering measurements of the on-chip silicon disk

**Fig. 4:**
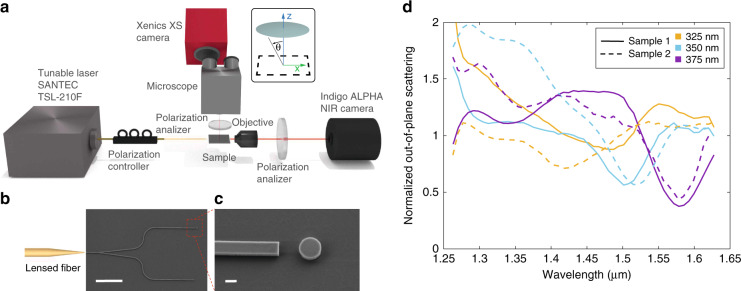
Far-field out-of-plane-scattering measurements. **a** Sketch of the experimental set-up. **b** SEM picture of one of the fabricated circuits, showing the input waveguide (with an external lensed fiber sketched), a Y-splitter, and two waveguides, both ended in an abrupt termination, and one of them having a disk (nominal disk radius *r* = 350 nm) close to the termination. Scale bar: 10 μm. **c** SEM image showing in detail the disk and the waveguide termination. Scale bar: 400 nm. **d** Experimental results of the out-of-plane scattering normalized to the value for a bare waveguide termination, recorded for disks with different radii (nominal values shown in the figure) in two different samples

To confirm these numerical predictions, two samples containing sets of waveguide-disk circuits were fabricated on a silicon-on-insulator chip using standard fabrication tools (see “Methods”). The far-field scattering in the out-of-plane direction was then measured using the experimental set-up that is schematically shown in Fig. 4a^31^. Note that this set-up allows us to inject the TE-like mode into the circuit—even though the waveguide is not single-mode—by measuring the polarization of the light at the output along the horizontal path and minimizing the presence of the TM-like mode (see details in “Methods”).

Each individual circuit (see Fig. 4b) includes a 3 mm long input waveguide, whose initial width (3 μm) is chosen to maximize the coupling efficiency to an external lensed fiber in an end-fire coupling approach, and which is adiabatically narrowed down to *w* = 420 nm. Then a 3-dB Y-splitter is inserted to split the input signal into two paths ended in an abruptly terminated waveguide, with a silicon disk patterned in only one of the paths. The microscope objective of the measurement system allowed us to record the field intensity scattered from the circuit in normal direction, separating the radiated emission from the regions highlighted with red squares in Supplementary Fig. S5. Notice that the use of an objective with finite numerical aperture will result in collecting light propagating inside a cone with half-opening angle of Δ*θ* ≈ 5^∘^ around the z-axis (*θ* = 0^∘^), thus producing a residual background coming from multipolar terms different to the electric and toroidal dipoles. Figure 4b, c shows scanning electron microscope (SEM) images of a fabricated circuit, highlighting in detail the waveguide end acting as an excitation port as well as the disk. Figure 4d shows the measured normalized out-of-plane scattering for three different disks having nominal radii of 325, 350, and 375 nm. Notice that the far-field scattering is not spatially tailored, in contrast to other integrated approaches such as the one demonstrated in ref. ^35^. The scattering responses, measured for two different samples containing circuits with identical nominal radii of the disks, are qualitatively similar to the ones obtained in simulation (Fig. 3d). In particular, there is a region with reduced scattering coincident with the predicted anapole condition. The scattering is, indeed, smaller than for the case of the terminated waveguide without disk (in other words, normalized scattering < 1, going even below 0.5 at some wavelengths, in agreement with numerical simulations), which means that not only the scattering is well suppressed, but also that the excitation of the electric dipole and toroidal moments corrects the divergence of the beam exiting the waveguide termination and contribute to reduce the overall scattering out of the chip. Note that, as mentioned above, the excitation field is not a plane wave. This fact, together with the presence of the substrate makes that the normalized scattering becomes lower than unity around the anapole condition, in contrast to the plane-wave illumination case when the residual normalized scattering would be equal to one. The differences in the position of the scattering minimum for the two samples is caused by the different radii in the fabricated samples. Indeed, the radius of the fabricated disks was about 5% smaller than the nominal values due to overetching (see details in “Methods”). However, a significant deviation to the scattering response shown in Fig. 3d is observed for the disk having a nominal radius *r* = 325 nm in Sample 1. Despite the estimated disk axes being only slightly different to the ones in Sample 2 (see “Methods”), we observe a significant red-shift of the scattering minimum. This scattering curve is also slightly top-displaced in comparison with the other responses. While we did not detect any large debris via SEM inspection, both observations together hint at potential minor fabrication irregularities or lithographic resist residue close to this specific disk being responsible for the higher scattering and spectral shift. Still, the possibility to tune the anapole state by changing the radius is clearly demonstrated from these measurements.

### Near-field measurements

**Fig. 5:**
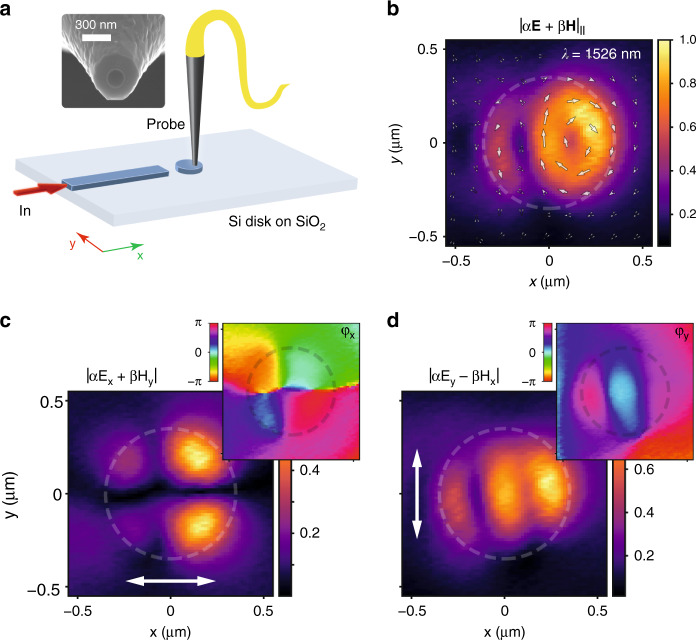
Phase- and polarization-resolved near-field measurements. **a** Sketch of the experimental set-up, utilizing an aperture-based near-field probe (see inset). **b** Near-field map of the normalized in-plane field amplitude as well as time-instantaneous field orientation (represented by white arrows) collected 30 nm above a silicon disk of nominal radius 375 nm (highlighted by the white dashed outline) at the wavelength of maximum near-field energy. **c** $\left|\leftrightarrow \right\rangle$ field map, consisting of a coherent sum of *E*_*x*_ and *H*_*y*_, with the detected phase distribution shown as inset. **d** $\left|\updownarrow \right\rangle$ field map, consisting of a coherent sum of *E*_*y*_ and − *H*_*x*_ as well as its phase distribution. The white arrows indicate the collected in-plane polarization direction. Both maps are normalized to the maximum field amplitude in **b**

In addition to the observed far-field scattering minimum, we experimentally verify the predicted dual-vortex structure of the anapole state as well as the numerically found decoupling of the minimum scattering condition and maximum-energy enhancement in the silicon disk by performing phase- and polarization-resolved near-field microscopy^36^ on one of the samples (Sample 2) used in the far-field measurements (see Fig. 4d). We utilize an aperture-based near-field probe consisting of an aluminum-coated tapered optical fiber (see inset in Fig. 5a) with an opening of 175 nm, and raster-scan the probe over the disk of nominal radius *r* = 375 nm from Sample 2 of Fig. 4d (actual radius measured via SEM: *r* = 355 nm), 30 nm above its top surface (see Fig. 5a, as well as “Methods” for additional details). With such a near-field probe collecting both, in-plane electric and magnetic field components^37^, we extract near-field amplitude maps of the excited mode structure in the silicon disk (see Fig. 5b for a wavelength of *λ* = 1526 nm). The response of the near-field probe to the electric and magnetic field components is here given by the coupling coefficients *α* and *β*, respectively (see “Methods” and ref. ^37^). Being able to not only detect the amplitude but also local phase and in-plane polarization information of the near field (Fig. 5c, d) furthermore allows us to visualize the time-instantaneous field distribution, shown as white arrows in Fig. 5b for one given snapshot in the field’s optical cycle. The extracted full near-field information confirms the predicted dual vortex in the polarization structure of the mode inside the silicon disk at wavelengths around the anapole condition (see Supplementary Fig. 6 for a direct comparison between experimentally retrieved and numerically calculated field distributions), while also highlighting the distinct difference to the symmetric field distribution of the anapole state under normal incidence.

**Fig. 6:**
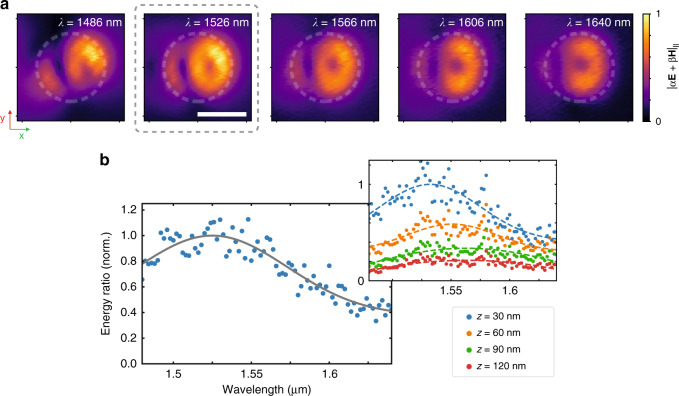
Wavelength-dependent near-field energy. **a** Near-field map of the in-plane field amplitude collected 30 nm above a silicon disk of nominal radius 375 nm (highlighted by the white dashed outline) in wavelength-steps of 40 nm around the point of maximum near-field energy (highlighted by a gray dashed box). The maps are normalized to the maximum field amplitude for the map of maximum near-field energy. Scale bar: 500 nm. **b** Spectral evolution of the near-field energy normalized to the emission of the feed waveguide. The gray line shows a Gaussian fit to the experimental data points. Inset: influence of the near-field probe on the spectral position of the energy maximum, highlighting the blue-shift when reducing the distance *z* between disk and probe^38,39^

Sweeping the excitation wavelength between 1480 nm and 1640 nm in steps of 1 nm and scanning the resulting field distribution, we extract the near-field energy above the disk’s top surface by integrating for each wavelength over the area of the disk and normalizing to the mode energy emitted from the feed waveguide (see Supplementary Section 1 for details). For the disk with nominal radius of *r* = 375 nm, we see that the near-field maps over the full probed wavelength range (Fig. 6a) exhibit the dual-vortex structure expected from the toroidal dipole moment, with the two vortex centers best visible at the wavelength of maximum energy at *λ* = 1526 nm, highlighted by a dashed gray line in the figure. The minor asymmetries observed in the collected field structures are an effect of the imperfect waveguide terminations, leading to a small tilt of the excitation wavefront as confirmed by numerical simulations. The energy ratio on top of the disk, normalized to its maximum value, is shown in Fig. 6b. The gray line corresponds here to a Gaussian fit to the experimental points shown in blue. The strong blue-shift of the maximum position with respect to the expected field maximum at *λ* ≈ 1600 nm for the investigated disk is here caused by the coupling between the near-field probe and the silicon disk^38,39^. We confirm this light-matter interaction by collecting the near-field energy in the center of the disk at different heights above the disk surface, resulting in a successive blue-shift of the maximum-energy wavelength and collected energy enhancement as the probe is brought closer to the disk (see inset in Fig. 6b). Comparing the experimentally determined wavelength maxima for different heights above the disk with numerical simulations (see Supplementary Fig. 7), we extract an unperturbed energy maximum at *λ* = 1600 nm, in excellent agreement with the numerical expectations.

## Discussion

We have shown that the excitation of a radiationless anapole state in a silicon disk exhibits remarkable differences when excited on chip, i.e., from the side, in comparison with the commonly used normal incidence approach. Interestingly, the anapole condition and the maximum field energy inside the disk occur at well separated wavelengths. We attribute this phenomenon to the large size of the disk in the excitation direction, so that retardation effects play a role in the multipolar decomposition. Besides numerical simulations, far- and near-field experiments confirm this observation. Measurement of far-field out-of-plane scattering confirm the existence of a wavelength-region with suppressed scattering around the predicted anapole wavelength. As expected from the simulations, the minimum scattering wavelength can be tuned by changing the disk radius. In our near-field measurements we confirm that the maximum concentration of energy inside the disk is red-shifted in comparison to the anapole condition. Still, we observe the local electric-field vortices, having different sense of rotation at each side of the disk, as expected from toroidal excitations. Notably, the two vortices change from being symmetric (in amplitude) at the anapole condition to highly asymmetric at the maximum-energy region. Similar phenomena might also occur for other states based on mode interference^12,40^ when excited in on-chip configuration. We believe our finding may have important consequences when trying to employ anapole states for sensing or nonlinear applications in on-chip nanophotonics^41^, either implemented in silicon or other high-index materials.

## Methods

### Numerical simulations

Multipole calculations in Fig. 1b, e, the spectra representing the energy inside the disk and on the disk surface, as well as the electric-field intensity at the disk center in Fig. 1c and f and the near-field maps in Fig. 2b have been performed by means of a finite-element method implemented in the commercial software COMSOL Multiphysics. In particular, we use the Radio Frequency Module that allows us to formulate and solve the differential form of Maxwell’s equations (in the frequency domain) together with boundary conditions. The disk is placed at the center of a spherical homogeneous region filled with air, whose radius is *λ*/2. A perfectly matched layer domain, with thickness *λ*/4, is positioned outside of the embedding medium domain and acts as an absorber for the scattered field. The mesh is chosen sufficiently fine as to allow numerical convergence of the results. In particular, the element size of the mesh of the embedding medium is smaller than *λ*/5 and that of the particles is smaller than *λ*/[3*ℜ*(*n*)], being *n* the silicon refractive index. The different multipolar contributions were obtained by integrating the displacement current induced inside the nanoparticle. The energy inside the disk corresponds to the volume average of the energy density time average at the disk volume. The energy on the disk surface was attained by means of the surface average of the energy density time average at the top surface of the disk.

Numerical results in Fig. 3d, e, Supplementary Figs. 2 and 4, as well as the scattering under waveguide illumination in Fig. 2a were obtained via 3D-FDTD simulations (RSoft tool by Synopsys). The spectra in Fig. 3d, e were obtained as the Fourier transform of the transverse electric field monitored either at a 2 µm distance from the disk center or at the disk center with and without disk to get the normalized response after exciting the TE mode of the waveguide with a pulsed signal centered at 1550 nm. Perfectly matched layers were applied at the boundaries of the simulation domain. The height to monitor the scattering, while not being truly far field, was chosen to avoid very time-consuming computations. In Supplementary Fig. 4, the energy in the silicon disk, in a cylinder (thickness 50 nm, radius equal to the silicon disk) placed 50 nm on top of the disk surface, and in a cylinder (thickness 50 nm, radius equal to the silicon disk) placed 2 µm on top of the disk surface were monitored for different wavelengths of the input TE mode. Scattering calculations in Supplementary Fig. 2 were performed by enclosing the disk in a cubic 2 × 2 × 2 µm^3^ box, launching a plane wave and measuring the near field on the surface of an enclosed volume. Then, the far field is computed from the recorded near field, which allows to obtain the scattering.

Numerical results in Fig. 3b, c and Supplementary Figs. 6 and 7 were obtained with Lumerical FDTD. The silicon disk, feed waveguide and silica substrate were enclosed by perfectly matched layers, while a mode source was used to launch a broadband wave in the feed waveguide. The fields on top of the disk were extracted by a field monitor, with the in-plane electric-field vectors for Fig. 3b, c selected at one time instant corresponding to the same phase of the *E*_*y*_ component at the center of the disk. Numerical results in Supplementary Fig. 3 as well as the Supplementary movies have been obtained using the commercial software CST Microwave Studio, which implements a finite integration technique. To model the silicon structures, a refractive index *n* = 3.45 has been considered. A hexahedral mesh with 10 cells per wavelength has been used. Open boundary conditions (perfectly matched layer) have been selected for external facets. The system has been considered to be surrounded by air. Field monitors have been used to observe the fields through and around the disk.

### Fabrication

The disk and waveguide structures were fabricated on standard silicon-on-insulator (SOI) chips with a top silicon layer thickness of 220 nm (resistivity *ρ* ~ 1 − 10 Ω cm^−1^, with a lightly p-doping of 10^15^ cm^−3^) and a buried oxide layer thickness of 3 µm. The fabrication is based on an electron beam direct writing process performed on a coated 100 nm hydrogen silsesquioxane resist film. The mentioned electron beam exposure, performed with a Raith150 tool, was optimized in order to reach the required dimensions employing an acceleration voltage of 30 keV and an aperture size of 30 µm. After developing the HSQ resist using tetramethylammonium hydroxide as developer, the resist patterns were transferred into the SOI samples employing an optimized Inductively Coupled Plasma- Reactive Ion Etching process with fluoride gases.

Two samples were fabricated, each one having three circuits with disks of nominal radius 325, 350, and 375 nm. The samples were characterized by SEM and the actual dimensions of the disk measured, with sample 1 used for far-field measurements in Fig. 4, and sample 2 for far-field measurements in Fig. 4 and near-field measurements in Figs. 5 and 6.

**Table:** 

	Sample 1			Sample 2		
Nominal disk radius *r*	325 nm	350 nm	375 nm	325 nm	350 nm	375 nm
SEM estimated *x*-axis	305 nm	320 nm	350 nm	297 nm	327 nm	355 nm
SEM estimated *y*-axis	310 nm	323 nm	359 nm	305 nm	332 nm	358 nm

### Far-field scattering measurements

Far-field scattering measurements were performed using the set-up depicted on Fig. 4a. Light was generated with a SANTEC TSL-210F laser, which is tunable in the wavelength range between 1260 nm and 1630 nm. A fiber polarizer was used to ensure that only a TE-like mode was excited in the input waveguide, which was carried out via a lensed fiber. The lensed fiber and the sample were placed in positioning platforms. Scattered light was captured with a stereoscope optical microscope (model 420-1105-10, National Optical & Scientific Instruments Inc. with effective numerical aperture NA ≈ 0.1 and high resolution, achromatic, color corrected lenses suitable for IR measurements) and detected in a Xenics XS infrared camera (model XS-1.7-320), which is equipped with a standard InGaAs detector that has a quantum efficiency between 85-100 % between 1250 and 1650 nm (100 % between 1450 and 1650 nm). An averaging of seven intensity measurements per wavelength was carried out to reduce the ripple in the measured scattering and then a smoothing of the data was performed by “polyfit” and “polival” Matlab functions, as previously done in ref. ^31^. At the output of the chip (along the horizontal path), a polarization filter was used to select the right mode in the waveguide by imaging onto an Indigo Alpha NIR camera for alignment purposes. In particular, the filter was placed vertically and the fiber polarizer was rotated until the power detected by the camera was zero, resulting in an estimated ratio of TE/TM-like modes of better than 100:1.

### Near-field optical measurements

Near-field measurements were performed in a home-build phase- and polarization-resolving near-field optical microscope^36^. The excitation light from a SANTEC TSL-710 laser was split into a signal and reference branch for interferometric phase detection, with the signal light being end-fire-coupled into the feed waveguide by a microscope objective. Free-space polarization control ensures that only the TE mode of the feed waveguide is excited, which is confirmed by measuring the mode dispersion of the waveguide close to the disk (see Supplementary Fig. 8 for details). The aperture-based near-field probe consists of a tapered single-mode optical fiber coated with 150 nm aluminum and an apex opening of 175 nm. The probe-to-sample distance is controlled via either shear-force feedback for scans on the surface of the sample, or optical height feedback via a SmarAct picoscale interferometer. The light collected through the fiber-based probe is combined with the reference beam in an optical fiber network, with the reference light frequency-shifted by 40 kHz to allow for heterodyne detection of the amplitude and phase of the collected signal. Polarization resolution is achieved by splitting the combined light beam into two orthogonal polarization components and separately detecting the amplitude and phase of the x- and y-component of the collected light field.

Owing to the near-field probe exhibiting an electric and magnetic response, the retrieved near-field signal is a combination of both, in-plane electric and magnetic near fields. The complex tensors *α* and *β* quantifying the system response to in-plane field components are to first order given by diagonal (*α*) and off-diagonal (*β*) position-independent matrices combining the similar local field components *E*_*x*_ and *H*_*y*_ to one detection channel as well as *E*_*y*_ and *H*_*x*_ to the orthogonal detection channel. For the chosen probe type, the coupling coefficients in these matrices are virtually identical, with *α*_*xx*_ = *α*_*yy*_ = *Z*_0_*β*_*xy*_ = *Z*_0_*β*_*yx*_, where *Z*_0_ is the impedance of free space^37^.

## Supplementary information

Supplementary information: Radiationless anapole states in on-chip photonics

Supplementary Movie S1

Supplementary Movie S2

Supplementary Movie S3

Supplementary Movie S4

Supplementary Movie S5

Supplementary Movie S6

Supplementary Movie S7

Supplementary Movie S8

Supplementary Movie S9

Supplementary Movie S10

Supplementary Movie S11

Supplementary Movie S12

## Data Availability

All data shown in the manuscript and supplementary information can be found at 10.4121/14233682.
