# Supplementary material for: Radiationless anapole states in on-chip photonics
Source: Light Sci Appl. 2021 Oct 4;10:204. doi: 10.1038/s41377-021-00647-x (PMC8490413; doi:10.1038/s41377-021-00647-x)
Supplement: Supplementary file 1 — Supplementary information: Radiationless anapole states in on-chip photonics [file 41377_2021_647_MOESM1_ESM.pdf]

Supplementary information: Radiationless anapole states in on-chip photonics

Evelyn Díaz-Escobar,[1] Thomas Bauer,[2] Elena Pinilla-Cienfuegos,[1] Ángela I. Barreda,[1,3] Amadeu Griol,[1] L. Kuipers,[2] and Alejandro Martínez[1]

[1]*Nanophotonics Technology Center, Universitat Politècnica de València, Camino de Vera s/n, 46022, Valencia, Spain*
[2]*Department of Quantum Nanoscience, Kavli Institute of Nanoscience, Delft University of Technology, 2600 GA Delft, Netherlands*
[3]*Institute of Applied Physics, Abbe Center of Photonics, Friedrich Schiller University Jena, Albert-Einstein-Str. 15, 07745 Jena, Germany*

SUPPLEMENTARY TEXT

1. CALIBRATION OF THE COLLECTED NEAR-FIELD ENERGY AND RELATIVE PHASE

In order to compensate for spectral variations in the detected near-field energy that are caused by a spectral modulation of the coupling efficiency to the feed waveguide as well as varying reflection from its termination, the collected energy over the silicon disk surface is normalized by the energy emitted from the feed waveguide. To this extend, the field distribution of the feed waveguide directly adjacent to the silicon disk is probed over a length of 6 µm for each wavelength, and its dispersion relation is extracted via Fourier transform along the direction of the waveguide. Due to the inherent phase detection in our near-field measurement scheme, we can here distinguish modes propagating in forward and backward direction (see also supplementary figure S8 for the experimentally retrieved dispersion in forward direction of one of the samples). By restricting the reciprocal space to only positive k-values or only negative k-values and integrating the absolute squared of the resulting field maps, the mode energy injected into the feed waveguide and reflected from its termination can be determined with the difference between the two, thus providing a measure for the amount of energy emitted by the waveguide termination.

In addition to the extracted energy normalization, this waveguide calibration also allows to determine the relative phase between the two detected orthogonal field components. By selecting the forward-propagating mode, the phase between the x- and y-component of the field is calibrated in reference to the analytical mode solution of the waveguide, with the two components $\pi/2$ out of phase with each other.

SUPPLEMENTARY FIGURES

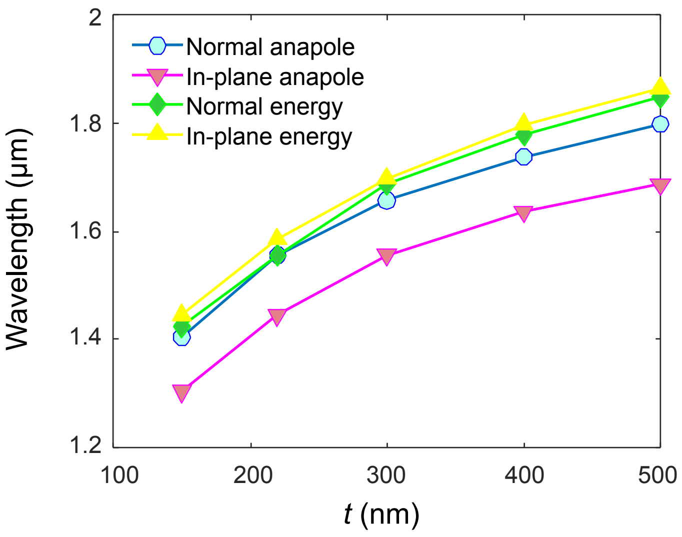

Figure S1. **Anapole-condition and maximum-energy wavelengths for normal and in-plane incidence** as a function of the disk thickness t for a fixed silicon disk radius r=350 nm.

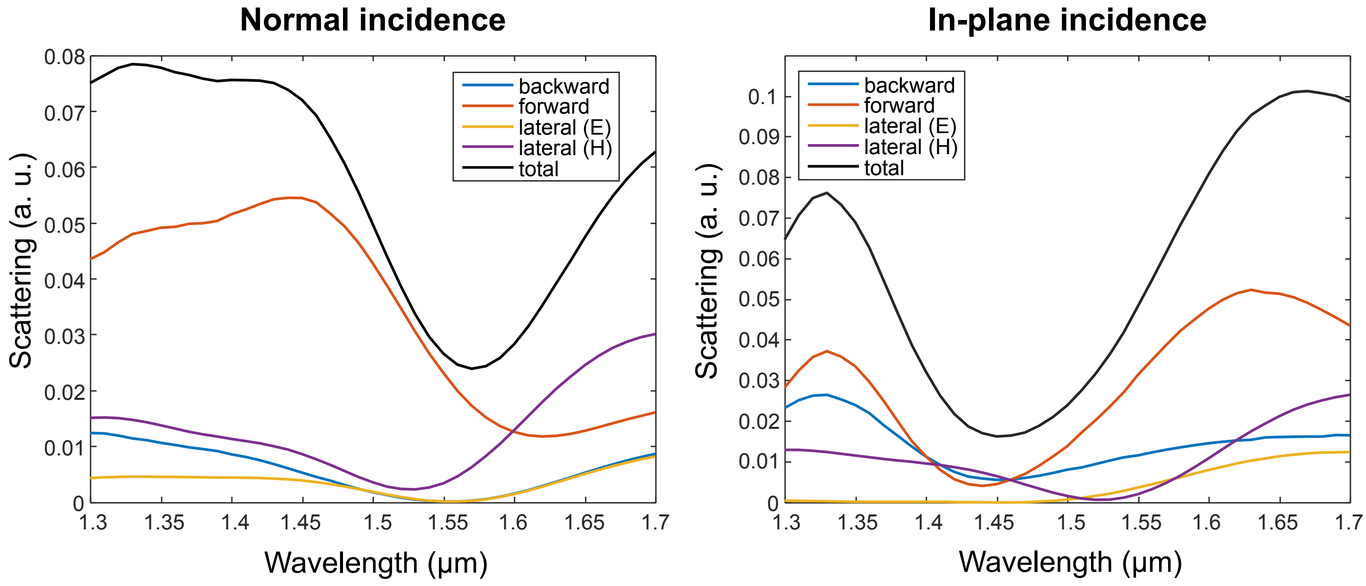

Figure S2. **Simulated scattering spectra for each configuration along different spatial directions for normal and in-plane plane-wave excitation of a silicon disk**. Lateral (E) and lateral (H) stands for the directions of the E and H field of the incoming wave, respectively. The disk dimensions are r = 350 nm and t =220 nm. This type of far field is computed from the near field on the surface of an enclosed volume and is valid for situations such as a field radiating from a point source and the scattered field from an enclosed plane wave launch. The results are only valid when the surface of the enclosed volume lies in a homogeneous region.

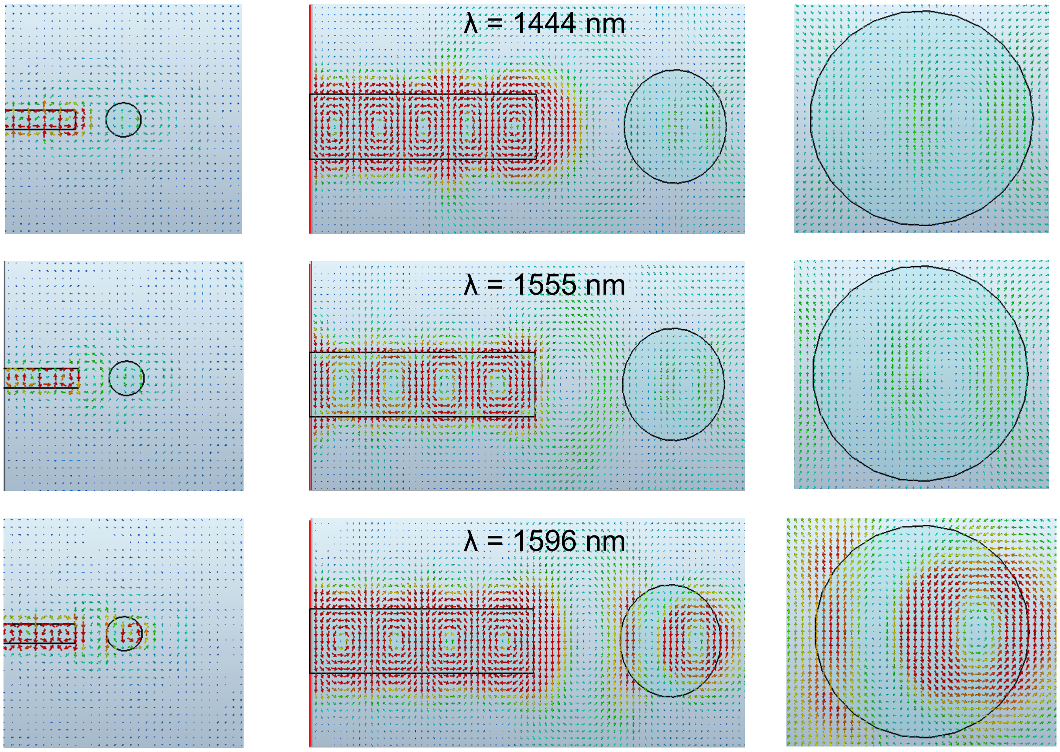

Figure S3. **Simulations of the electric field lines (represented by arrows) of a disk illuminated from a waveguide at three representative wavelengths**: anapole state for in-plane illumination (top row), anapole state for normal illumination (middle row) and energy maximum under in-plane illumination (bottom row). These results have been obtained using the commercial software CST Microwave Studio.

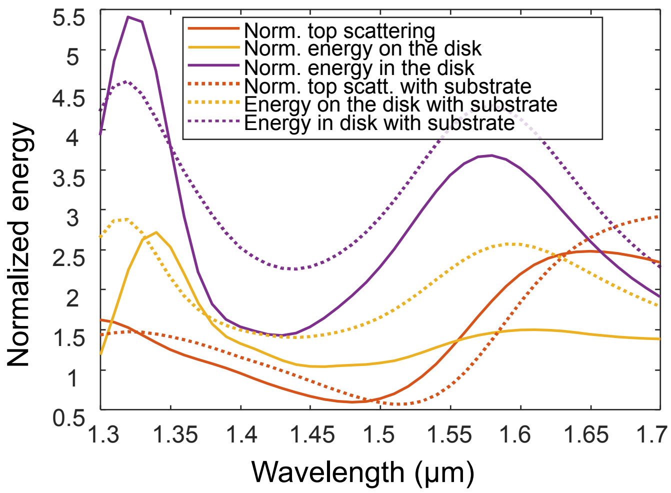

Figure S4. **Numerically calculated (using 3D-FDTD) energy inside the disk ($r = 350\,\text{nm}$), at $50\,\text{nm}$ spacing on top of the disk and at $2\,\mu\text{m}$ distance from the disk with and without silica substrate**. The latter metric is considered to be a good approximation of the scattered field. Illumination is in-plane from the waveguide end excited by the TE-like mode.

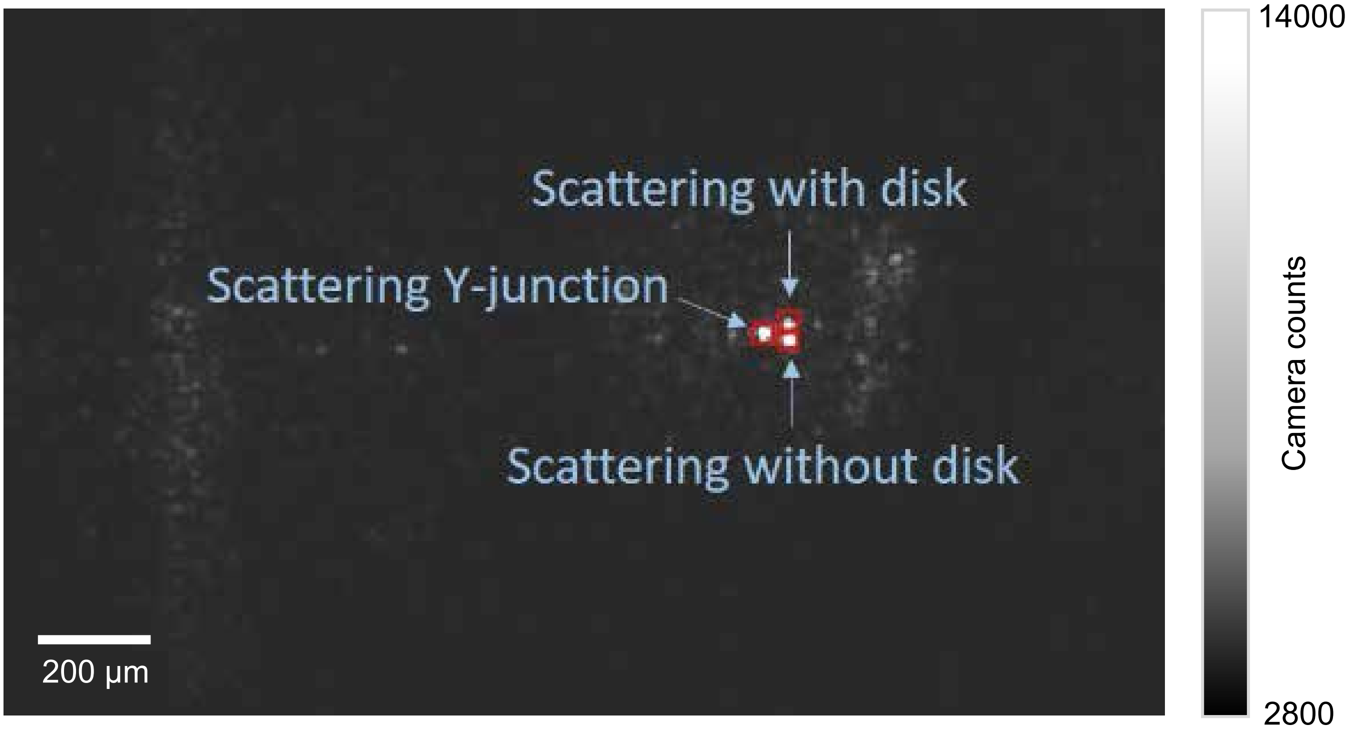

Figure S5. **Image recorded with the infrared camera in the far-field measurements** showing the spot corresponding to the Y-splitter, the waveguide termination with disk and the waveguide termination without disk. The scale bar in terms of camera counts is also depicted.

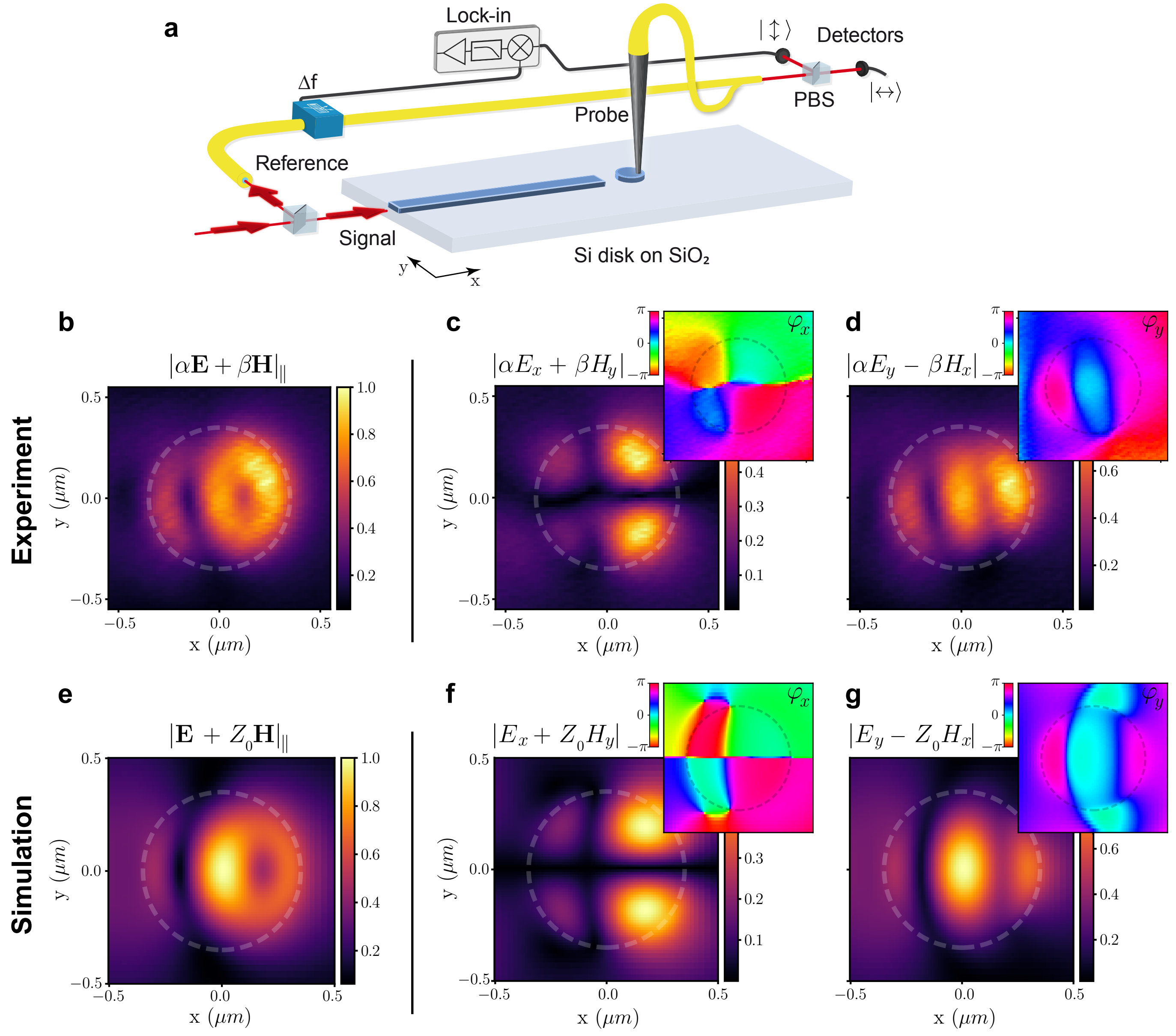

Figure S6. **Detailed experimental results of the phase- and polarization-resolved near-field optical microscopy (NSOM) on a** $r = 355\,\text{nm}$ **silicon disk under in-plane illumination**. **a**, Sketch of the NSOM setup, including the phase- and polarization-detection capabilities given by a heterodyne detection scheme via an acousto-optic modulator shifting the reference light frequency by $\Delta f = 40\,\text{kHz}$ and a polarization beam splitter (PBS) in the detection path. **b**, Experimentally retrieved in-plane field distribution at the wavelength of highest field energy ($\lambda = 1526\,\text{nm}$), normalized to its maximum value. The aperture-based near-field probe results in a collection of both, electric and magnetic field components. **c**, $|\leftrightarrow\rangle$ field distribution, consisting of a coherent sum of E_x and H_y, with the detected phase distribution shown as inset. **d**, $|\updownarrow\rangle$ field distribution, consisting of a coherent sum of E_y and $-H_x$ as well as its phase distribution. **e**, Numerically calculated in-plane field distribution 30 nm above the surface of a $r = 350\,\text{nm}$ silicon disk at the wavelength of highest field energy ($\lambda = 1596\,\text{nm}$, normalized to its maximum value. In analogy to the experimental collection, both electric and magnetic field components are considered and weighted with equal amplitude. **f**, $|\leftrightarrow\rangle$ field distribution, consisting of a coherent sum of E_x and $Z_0 H_y$, with the calculated phase distribution shown as inset. **g**, $|\updownarrow\rangle$ field distribution, consisting of a coherent sum of E_y and $-Z_0 H_x$ as well as its phase distribution. Z_0 corresponds here to the impedance of free space, and the white and gray dashed lines correspond to the outline of the investigated silicon disk. The calculations were conducted via Lumerical FDTD.

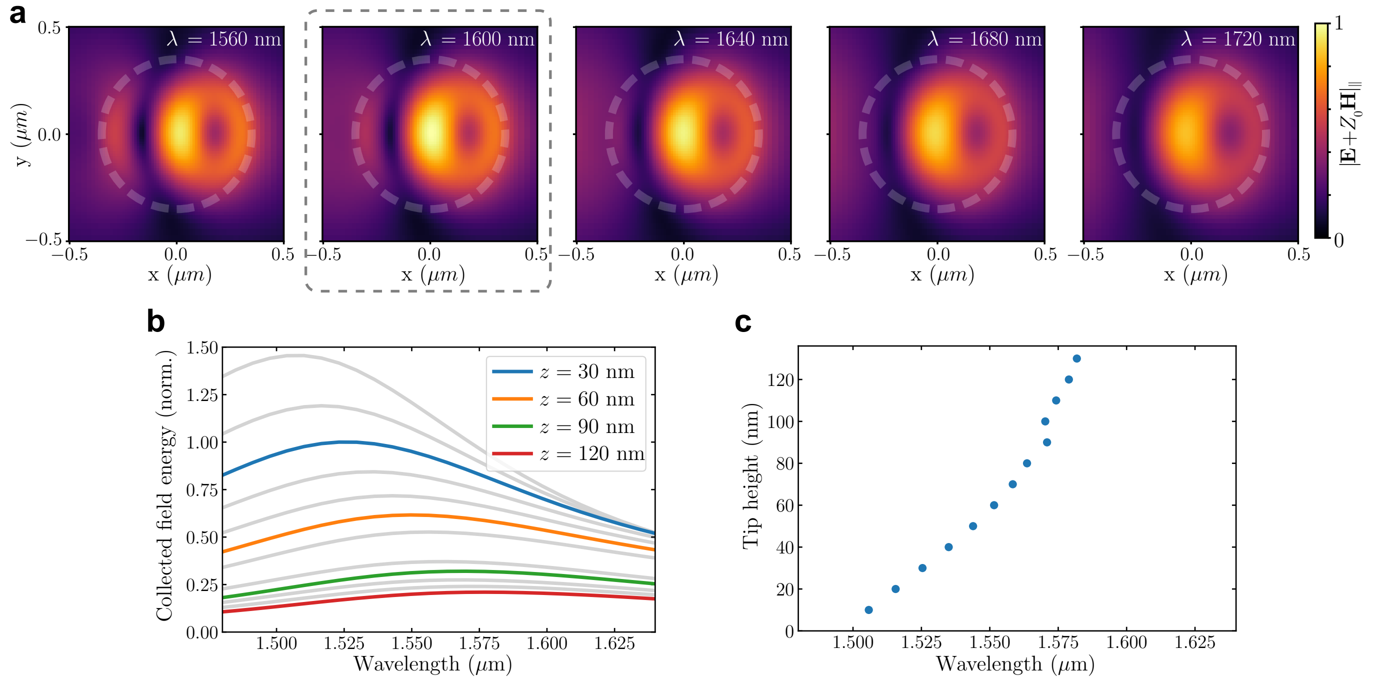

Figure S7. **Near-field optical detection simulated via FDTD calculations**. **a**, Near-field map of the in-plane field amplitude collected 30 nm above a silicon disk of radius 350 nm (highlighted by the white dashed outline) in wavelength-steps of 40 nm around the point of maximum near-field energy (highlighted by a gray dashed box). The extracted in-plane electric and magnetic fields $\mathbf{E}_{\parallel}$ and $Z_0\mathbf{H}_{\parallel}$ are weighted with equal amplitude to simulate the effect of the near-field collection, with Z_0 corresponding to the impedance of free space. The maps are normalized to the maximum field amplitude for the map of maximum near-field energy. **b**, Calculated influence of the near-field probe on the spectrum of the energy enhancement. A tapered glass cone (taper angle $\alpha = 30°$) coated with 150 nm aluminium and an aperture of 175 nm is used as probe, while the energy flux is extracted inside the glass cone 2 μm above the aperture. The spectral response is shown in z-distance steps of 10 nm, with heights corresponding to the experimental realization in Fig. 6 of the main manuscript highlighted in colors. **c**, Extracted wavelength of the maximum energy enhancement for different tip-sample distances from 10 nm to 130 nm, verifying the measured spectral blue-shift of the maximum with decreasing distance.

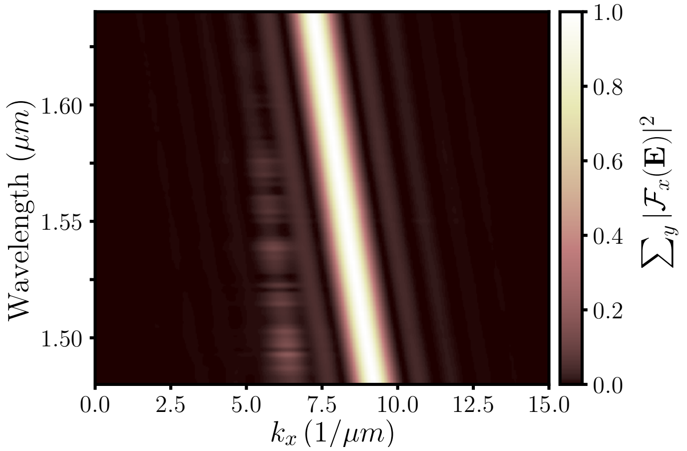

Figure S8. **Experimental determination of the TE/TM-like mode dispersion in the feed waveguide of the disk with nominal radius of** 375 nm. Utilizing the phase resolution of the near-field setup, we extract the field distributions of the modes propagating in the waveguide 20 nm above its surface, at a distance of 5 µm from the disk. A Fourier transform of the resulting complex amplitude field separates the TE-like mode from the TM-like mode due to their different mode indices, leading to two distinct dispersion relations. The dominant mode at higher in-plane wavenumbers corresponds here to the desired TE-like mode, while an additional TM-like mode at lower in-plane wavenumbers is visible for wavelengths up to ca. 1.6 µm. The ratio of the two modes is on average 20:1, with the intensity in the TM-like mode being at maximum 0.2 of the TE-like mode at specific wavelengths. The side lobes to the TE-like mode are here caused by the limited measurement length of the near-field scan taken to extract the dispersion.

Movie S1

Time-evolution of the absolute value of the in-plane electric field distribution when exciting a $r = 350\,\mathrm{nm}$ silicon disk from a feed waveguide in free space at the anapole condition ($\lambda = 1444\,\mathrm{nm}$). Collection plane: center of the disk.

Movie S2

Time-evolution of the absolute value of the in-plane electric field distribution when exciting a $r = 350\,\mathrm{nm}$ silicon disk from a feed waveguide in free space at the anapole condition ($\lambda = 1444\,\mathrm{nm}$). Collection plane: on top of the disk.

Movie S3

Time-evolution of the absolute value of the in-plane electric field distribution when exciting a $r = 350\,\mathrm{nm}$ silicon disk from a feed waveguide in free space at the normal-incidence anapole condition ($\lambda = 1555\,\mathrm{nm}$). Collection plane: center of the disk.

Movie S4

Time-evolution of the absolute value of the in-plane electric field distribution when exciting a $r = 350\,\mathrm{nm}$ silicon disk from a feed waveguide in free space at the normal-incidence anapole condition ($\lambda = 1555\,\mathrm{nm}$). Collection plane: on top of the disk.

Movie S5

Time-evolution of the absolute value of the in-plane electric field distribution when exciting a $r = 350\,\mathrm{nm}$ silicon disk from a feed waveguide in free space at the wavelength of maximum energy inside the disk ($\lambda = 1596\,\mathrm{nm}$). Collection plane: center of the disk.

Movie S6

Time-evolution of the absolute value of the in-plane electric field distribution when exciting a $r = 350\,\mathrm{nm}$ silicon disk from a feed waveguide in free space at the wavelength of maximum energy inside the disk ($\lambda = 1596\,\mathrm{nm}$). Collection plane: on top of the disk.

Movie S7

Time-evolution of the y-component of the electric field distribution when exciting a $r = 350\,\mathrm{nm}$ silicon disk from a feed waveguide in free space at the anapole condition ($\lambda = 1444\,\mathrm{nm}$). Collection plane: center of the disk.

Movie S8

Time-evolution of the y-component of the electric field distribution when exciting a $r = 350\,\mathrm{nm}$ silicon disk from a feed waveguide in free space at the anapole condition ($\lambda = 1444\,\mathrm{nm}$). Collection plane: on top of the disk.

Movie S9

Time-evolution of the y-component of the electric field distribution when exciting a $r = 350\,\mathrm{nm}$ silicon disk from a feed waveguide in free space at the normal-incidence anapole condition ($\lambda = 1555\,\mathrm{nm}$). Collection plane: center of the disk.

Movie S10

Time-evolution of the y-component of the electric field distribution when exciting a $r = 350\,\mathrm{nm}$ silicon disk from a feed waveguide in free space at the normal-incidence anapole condition ($\lambda = 1555\,\mathrm{nm}$). Collection plane: on top of the disk.

Movie S11

Time-evolution of the y-component of the electric field distribution when exciting a $r = 350\,\mathrm{nm}$ silicon disk from a feed waveguide in free space at the wavelength of maximum energy inside the disk ($\lambda = 1596\,\mathrm{nm}$). Collection plane: center of the disk.

Movie S12

Time-evolution of the y-component of the electric field distribution when exciting a $r = 350\,\mathrm{nm}$ silicon disk from a feed waveguide in free space at the wavelength of maximum energy inside the disk ($\lambda = 1596\,\mathrm{nm}$). Collection plane: on top of the disk.
